# Factors affecting hospital stay in psychiatric patients: the role of active comorbidity

**DOI:** 10.1186/1472-6963-12-166

**Published:** 2012-06-19

**Authors:** Athanassios Douzenis, Dionysios Seretis, Stella Nika, Paraskevi Nikolaidou, Athanassia Papadopoulou, Emmanouil N Rizos, Christos Christodoulou, Christos Tsopelas, Dominic Mitchell, Lefteris Lykouras

**Affiliations:** 1 Second Psychiatry Department, Athens University Medical School, Attikon General Hospital, 1 Rimini st, Athens, 12462, Greece; 2 Department of Psychology, 2 South, University of Bath, Bath, BA2 7AY, UK; 3 Psychiatric Hospital of Attica, 374 Athinon Ave, Chaidari, Athens, 12462, Greece; 4 Department of Computer Science, East building, University of Bath, Bath, BA2 7AY, UK

**Keywords:** Bipolar, Schizophrenia, Referral, Hospital stay, Physical comorbidity

## Abstract

**Background:**

Research on length of stay (LOS) of psychiatric inpatients is an under-investigated issue. In this naturalistic study factors which affect LOS of two groups of patients were investigated, focusing on the impact on LOS of medical comorbidity severe enough to require referral.

**Methods:**

Active medical comorbidity was quantified using referral as the criterion. The study sample consisted of 200 inpatients with the diagnosis of schizophrenia and 228 inpatients suffering from bipolar disorder (type I or II). Jonckheere and Mann–Whitney tests were used to estimate the influence of referrals on LOS, and regression analyses isolated variables associated with LOS separately for each group.

**Results:**

Half of the patients needed one or more referrals for a non-psychiatric problem. The most common medical condition of patients with bipolar disorder was arterial hypertension. Inpatients with schizophrenia suffered mostly from an endocrine/metabolic disease - 12% of referrals were for Hashimoto’s thyroiditis. A positive linear trend was found between LOS and number of referrals; the effect was greater for schizophrenia patients. The effect of referrals on LOS was verified by regression in both groups. Overall, referred patients showed greater improvement in GAF compared to controls.

**Conclusions:**

To our knowledge this was the first study to investigate physical comorbidity in psychiatric inpatients using the criterion of referral to medical subspecialties. Comorbidity severe enough to warrant referral is a significant determinant of hospital stay. This insight may prove useful in health care planning. The results show lack of effective community care in the case of schizophrenia and negative symptoms may be the cause of this. Our findings call for more attention to be paid to the general medical needs of inpatients with severe mental health and concurrent severe medical comorbidity.

## Background

Mental health patients often suffer from concurrent medical conditions [1]. Rates of medical morbidity are reported to be particularly high in certain groups of patients. It has been estimated that more than half of patients with schizophrenia suffer from a chronic medical problem [2].There are reports of increased rates of hypertension [3], hyperlipidemia, obesity and diabetes [4,5]. Patients suffering from bipolar disorder also report increased medical comorbidity [6] with asthma, chronic bronchitis, hypertension, and gastric ulcer [7] as well as diabetes, coronary artery disease and dyslipidemia [8].

Instances of physical medical problems have been associated with an increased burden on psychiatric patients resulting in poorer outcomes for their psychiatric condition, greater severity of psychiatric symptoms [9] as well as an increased incidence of non-compliance with treatment. In this respect, the prompt identification of comorbid physical problems for mental health patients is a significant issue that might have a role in improving subsequent outcomes, both medical and psychiatric. Regarding inpatients there is an additional reason for addressing physical problems. It has been suggested that medical comorbidity increases the length of stay (LOS) for psychiatric inpatients either directly, by increasing psychiatric symptoms or indirectly by demanding the focus of medical attention during hospitalization [10].

Longer admission, of course, means a greater financial burden incurred. The last fifty years have been witness to a significant change in mental health services delivery. Attention has focused on using less and less inpatient treatment, replacing hospital stay with treatment in the community. In fact, longer hospital stay may nowadays imply poor mental health care and support in the community. As a consequence, during the last two decades there has been an increased interest by administrators and governments responsible for financing mental health services in reducing the money spent on inpatient services and consequently in LOS reduction. Reduction of LOS is associated with less expenditure and reducing LOS is considered to be a sign of successful treatment in the community.

Hospital stay has mostly been investigated with consecutively admitted inpatients irrespective of diagnosis perhaps because early studies showed that diagnosis is not associated with LOS [11,12] an issue which remains controversial. Findings suggest that prediction of LOS is far from straightforward given the complex nature of LOS and the multiplicity of the factors involved [13-15]. Physical comorbidity is one aspect which has not been investigated enough in reference to LOS. Comorbidity can prove to be a nebulous concept and this has been a complicating factor in the study of its effects. On the one hand there are individuals who have a physical problem (for instance hypertension) they are aware of and receive treatment for and on the other, there are individuals whose physical ailment is discovered during the admission for treatment of another disorder. It is obvious that the latter patients require more attention and need assessment and treatment for their physical illness urgently and effectively. The distinction between serious ‘active’ medical comorbidity that receives medical attention and ‘passive’ comorbidity was described by Lyketsos et al. [10].

In this study we quantified the concept of active medical comorbidity using referral as the criteria. The primary objective was to investigate whether and to what extent the physical comorbidity that required medical attention increased the LOS of inpatients with schizophrenia or bipolar disorder (type I or II). We measured severe active comorbidity for which one, two, three or more referrals were made to other specialties by the attending psychiatrist. A passive comorbid condition was one that after admission did not need referral to another medical specialty for modification of treatment either because it was existent and known to the patient prior to admission or because it was discovered during admission and was pharmacologically (or otherwise) controlled by the attending psychiatrist. An active comorbid condition was one that needed referral to a medical specialty during hospitalization.

Our study had the following aims: 1) To explore the prevalence and range of comorbid conditions for which referral was needed in patients suffering from schizophrenia and bipolar disorder. 2) To test the hypothesis that severe comorbidity requiring referral would affect length of stay in a linear fashion, in both groups of patients 3) To investigate which factors, including referral, are associated with length of stay across both diagnostic groups. 4) To investigate whether referrals had an impact on patients’ ability to function as measured by the scale General Assessment of Functioning.

## Results

### Schizophrenia group

We calculated the number of medical-surgical problems for which patients with schizophrenia were referred to other medical specialties AND subsequently received treatment for, in absolute number and in terms of percentage of patients within the schizophrenia group. 100 patients (50%) had no referral for a comorbid condition and 100 patients (50%) had one or more referrals. Of the latter 58 patients (29% of patients with schizophrenia) had one referral, 24 patients (12%) had two referrals and 18 patients (9%) had three or more referrals.

Table 1 shows the prevalence in % percentages of ICD-10 categories as well as specific diagnoses of physical conditions which needed referral to specialists across the schizophrenia group.

**Table 1:** Prevalence of physical conditions which needed referral

	**Endocrine**	**Circulatory**	***Nervous***	**Respiratory**	**Musculoskeletal**	**Blood**	**Skin**
**Sch.: % ICD-10**	24%	20%	3%	9%	2%	1%	5%
**Sch.: % diseases**	Hashimoto’s thyroiditis 12% Non-insulin-dependent diabetes mellitus 3%	Other conduction disorders 14% Arterial hypertension 6%	Vascular syndromes of brain in cerebro vascular diseases 1%	Acute nasophangitis 4% Unspecified chronic bronchitis 2%	Pain in joint 1%	Iron deficiency anaemia 1%	Infective dermatitis 1%
**BD: % ICD-10**	13%	23.7%	4.4%	7%	1.7%	4.4%	7%
**BD: % diseases**	Congenital hypothyroidism with diffuse goitre 3.5% Hashimoto’s thyroiditis 2.6%	Arterial hypertension 15% Other conduction disorders 7%	Parkinson’s disease 1.7%	Unspecified chronic bronchitis 1.76% Asthma 1.76%	Systemic lupus erythematous 0.9%	Iron deficiency anaemia 3.5%	Atopic dermatitis 2.6%

Abbreviations: *Sch* Schizophrenia, *BD* Bipolar disorder.

The median LOS for schizophrenia patients was 18 days (Mean 19.57 SD 11.227 N 200).

The Jonckheere Test was run to test for a linear association between number of referrals and LOS. All four groups of patients were compared (no referral, one referral, two referrals, three or more referrals). The test showed a significant linear trend in the data *J* = 8264, z = 4.210, r = .29 p < .001 indicating that as the referrals increased so did LOS. (Asymptotic 1-tailed sig. are reported throughout the results section unless stated otherwise).

Post-hoc Mann–Whitney tests were run to test for differences in LOS between the three conditions (one referral, two referrals, three or more referrals) and the control group (no referral). A Bonferroni correction was applied and the significance level was set at .0166. Table 2 shows the results across groups for schizophrenia patients. Two-Sample Kolmogorov-Smirnov Tests are also reported when any one of the subgroups had small number of participants. There were statistically significant differences between each of the three subgroups (one, two and three or more referrals) and the control group (no referral).

**Table 2:** Descriptive statistics and post-hoc tests across groups of referrals

	**Number referrals**	**Median**	**Mean (SD)**	U*	**Exact Sig.**	Z**	**Exact Sig.**
**Schizophrenia**	0 referrals	14	16.46 (9.615)				
	1 referral	18	21.52 (12.136)	2230^a^	0.008^a^		
	2 referrals	19.5	20.17 (8.535)	852^b^	0.013^b^	1.657^b^	0.002^b^
	3 referrals	27	29.78 (12.804)	374^c^	0.000^c^	1.901^c^	0.000^c^
**Bipolar**	0 referrals	14	15.44 (7.641)				
	1 referral	14.5	16.25 (8.412)	4614^a^	0.364^a^		
	2 referrals	19	23.17 (11.111)	756^b^	0.001^b^	1.559^b^	0.003^b^
	3 referrals	19	22.50 (10.071)	252^c^	0.024^c^	1.011^c^	0.082^c^
**Both groups**	0 referrals (n = 208)	14	15.93 (8.640)				
	1 referral (n = 146)	15	18.34 (10.346)	13502^a^	0.038^a^		
	2 referrals (n = 48)	19.5	21.67 (9.917)	3158^b^	0.000^b^		
	3 referrals (n = 26)	26	27.54 (12.313)	1234^c^	0.000^c^		

**Symbols**^**a**^ no referral vs. one referral ^**b**^ no referral vs. two referrals ^**c**^ no referral vs. three or more referrals * U statistic of Mann Whitney test ** Z statistic of Two-Sample Kolmogorov-Smirnov test.

Thus, overall, LOS was increased in all three cases (one referral, two, three or more) and the more the referrals the greater the increase in LOS.

A standard multiple regression gave a significant fit to the model F [8] =14.561, p < .001). Results showed that predictors account for .381 of the variation in LOS, R = 0.62 R^2^ =0.381 Adjusted R^2^ = 0.355. All assumptions were met. Table 3 shows the effects of individual contributors on the outcome.

**Table 3:** Standard multiple linear regression: Individual predictors of hospital stay for schizophrenia and bipolar patients

		**Unstandardized coefficients**		**Stand coeff.**	**t**	**Sig.**	**95.0% CI for B**	
**Model for schizophrenia**		**B**	**Std. error**	**Beta**			**Lower bound**	**Upper bound**
	(Constant)	4.189	4.015		1.043	0.298	−3.73	12.108
	Gender	5.824	1.482	0.258	3.930	0.001	2.900	8.747
	On occupation or not during 6 months prior to hospitalization	4.702	1.485	0.191	3.166	0.002	1.773	7.631
	Substance misuse prior to hospitalization	−14.665	3.209	−0.600	−4.57	0.001	−20.995	−8.335
	The psychiatric comorbid status	16.956	3.414	0.687	4.966	0.001	10.222	23.691
	Age of onset of mental disorder	−0.314	0.058	−0.350	−5.418	0.001	−0.429	−0.200
	BPRS	0.234	0.048	0.291	4.928	0.001	0.140	0.328
	One ref vs. no ref	3.136	1.476	0.127	2.125	0.035	0.225	6.047
	Three ref vs. no ref	17.343	2.404	0.444	7.215	0.01	12.601	22.085
**Model for bipolar patients**		**B**	**Std. error**	**Beta**			**Lower bound**	**Upper bound**
	(Constant)	17.295	1.303		13.275	0.001	14.727	19.862
	Two ref vs. no ref	7.457	1.823	0.262	4.090	0.001	3.864	11.049
	Single vs. married or divorced*	1.827	0.607	0.190	3.011	0.003	0.631	3.022
	Distance from hospital**	−3.239	1.354	−0.151	−2.393	0.018	−5.907	−0.571
	Three ref vs. no ref	6.721	2.961	0.142	2.27	0.024	0.886	12.557

Symbols * binary variable ** binary variable: in Attica vs. Out of Attika.

### Bipolar group

As before, the number of medical problems for which patients with bipolar disorder were referred were calculated in absolute number and in terms of percentage of patients within the group. 108 patients (47, 4%) did not need referral. 120 patients (52, 6%) needed one or more referrals. Of these 88 patients (38, 6% within the bipolar group) had one referral 24 patients (10, 5%) had two referrals and only 8 patients 3, 5% had three or more referrals.

Table 1 shows the prevalence in percentages of ICD-10 categories and diagnoses of physical conditions which needed referral to specialists across the bipolar group.

The median LOS for patients with bipolar disorder was 15 days (Mean 16.82 SD 8.758 N 228), significantly lower than the schizophrenia group U = 19828, p (2-tailed) = .020 r = −.11.

The Jonckheere Test was run to test for a different effect of LOS among patients suffering from bipolar disorder (no referral, one referral, and two referrals, three or more referrals). The test showed a significant positive trend in the data J = 9410, z = 2.71, p = .003 r = .18.

Post-hoc Mann–Whitney tests were run to test for differences in LOS between three conditions as in the schizophrenia group. A Bonferroni correction was applied and the significance level was set at .0166. Again, Two-Sample Kolmogorov-Smirnov Tests are also reported. As shown in Table 2 only the patients with two referrals had a statistically significant prolonged LOS in comparison with the control group.

Thus, for bipolar patients the increase in LOS was positively associated with increase in number of referrals but only when two referrals were carried out, in this case severe active comorbidity actually had a significant impact on LOS.

A standard multiple regression for bipolar patients gave a significant fit to the model F [4] =8.934 p < .001. The variation in LOS, R = 0.372 R^2^ =0.138 Adjusted R^2^ = 0.123. All assumptions were met. Table 3 shows the effects of individual contributors on the outcome.

### Both groups

Applied to the whole sample the Jonckheere Test again showed a positive linear relationship between number of referrals (four levels) and LOS J = 35782, z = 5.071 p < .001 r = .25. Table 2 shows statistics. The effect on LOS was significant for patients with two referrals and patients with three or more referrals (r = −.25 and r = −.3 respectively).

**Figure 1:**
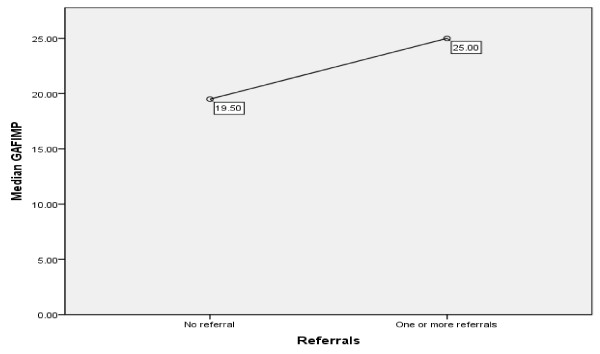
The effect of referrals on GAF improvement for the whole sample.

In addition, the overall effect of one or more referrals on the level of functioning was investigated, patients with no referral served as the control group. The Mann–Whitney test (based on the binary variable referral-non referral) was significant U = 19462, p (two tailed) = .007 r = −.13 indicating that patients who were referred to other specialties (n = 220) had a higher improvement in level of functioning as measured by GAF (GAF discharge - GAF admission) than patients with no active medical comorbidity (Figure 1).

## Discussion

### Comorbidity rates

In our study, 50% of the patients with schizophrenia and 52, 6% of patients with bipolar disorder needed referral for a physical illness. This percentage is considerably higher than the one reported by Lyketsos et al. where medical comorbidity which merited medical attention was present in 20.6% of the sample.

One in four inpatients with schizophrenia suffered from an endocrine/metabolic disease with 12% of referrals revealing Hashimoto’s thyroiditis. Patients suffering from schizophrenia often present with high rates of obesity, high blood pressure and diabetes, the so called metabolic syndrome [16-18]. There are studies that link endocrine disorders (including metabolic syndrome) to the use of antipsychotics [19-21]. However, the finding that 12% of the patients with schizophrenia were diagnosed with Hashimoto’s disease merits further attention as this percentage is higher than that usually reported in schizophrenia [22-24]. It has been argued that Hashimotos disease can be a possible endophenotype of bipolar disorder but to our knowledge, it has not been directly associated with schizophrenia [25].

### Length of stay of patients with schizophrenia

Most previous studies have studied inpatients with more than two diagnoses and several relied exclusively on admission markers of LOS. Thus, the findings of this study are not directly comparable with findings of earlier studies. Variability in designs, settings and samples further complicates matters. However, some variables that are common predictors of LOS are replicated here in the schizophrenia group.

For patients with schizophrenia severe comorbidity had a significant linear positive association with LOS. For all three sub-groups of patients LOS was significantly prolonged: medians of 4, 5.5 and 13 days more for the three groups (one ref, two refs, three or more ref) compared to patients with no referral. The regression analysis verified the positive effect on LOS. This is in line with Lyketsos et al. who discriminated between active and passive comorbidity, but interestingly not in accordance with the two studies which specifically focused on comorbidity and LOS [26,27] and found that in patients with schizophrenia comorbidity and LOS are not associated. Moreover, although referral (three vs. no) had the most salient effect in explaining the variance on LOS it appears that in this group of patients both demographic and clinical variables contribute substantially to increased stay. In concordance with similar studies we found that gender [28,29] and BPRS-E [30,31] account for differences in LOS. One plausible explanation for the extended stay is that patients suffering from schizophrenia do not receive proper treatment in the community and both physical and psychiatric problems are not optimally handled without submission to a general hospital psychiatric department This consequently leads to increased hospital stays and expenditure. This is alarming and needs urgent attention since there are indications that this is not unique to the Greek health system [2].

### Length of stay of patients with bipolar disorder

For patients with bipolar disorder, a positive significant trend was found between referral-requiring comorbidity and LOS. In comparison with the control group (no referral) no other group had a significant difference in LOS except for the group with two referrals, a median of 5.5 days more. P values suggest that a bigger sample might have been able to reach statistical significance for the other groups as well. Regression also showed that severe active comorbidity prolongs hospital stay. These results are only partly in line with Sloan et al. [26] and Schubert et al. [27] who found increased LOS for depressed patients as no significant differences were reported for not depressed bipolar patients. What is of significance, here, is that only sociodemographic variables had an impact on LOS. This was an unexpected and difficult-to-explain effect. Perhaps larger sample sizes and other factors need to be studied regarding the LOS of patients with bipolar disorder in order to be able to reach safer results for patients with this diagnosis as it is implausible that clinical factors such as severity of the psychiatric condition do not have an effect on hospital stay. In essence, the difference between the two groups in the influence of severe comorbidity and LOS can be seen as an indication that patients with bipolar disorder may receive better physical care whilst living independently. This can be attributed to the nature of their disorder (absence of negative symptoms etc.). Thus, the results appear to confirm previous evidence that negative symptoms can accurately predict LOS [30].

### Length of stay of the whole sample of patients

Analysis of the whole sample of inpatients showed that the level of functioning improves more in psychiatric inpatients with active comorbidity -when this is addressed- than in patients without. This finding proves the significance of meeting the needs of patients in terms of general medical conditions. Longer admissions one can argue, might lead to a greater reduction of psychopathology, however the increased length of admission (maximum 10 days) is not long enough to support the argument that pharmacotherapy is responsible for this improvement.

### Implications for health services

It is clear that provision of efficient community care for patients with schizophrenia is an issue of priority as it seems to prevent longer stays- and leads to better quality of life [32].

Moreover, given the elevated active comorbidity for both groups, the results confirm that general hospitals are a better investment of the public money than psychiatric units [32]; not least because when these patients are admitted to a psychiatric hospital or other inpatient psychiatric unit, their physical health needs are either not met or if addressed lead to even longer admission and greater cost. In the new era of short admissions our paper offers support to the argument that there is an additional reason for addressing quickly and effectively the medical problems of patients, since medical comorbidity resolution may influence good psychiatric outcome. To this end close cooperation between psychiatrists and medics is required which should be promoted not only at a senior level but also at the level of trainees and hospital interns [33]. Longitudinal research is needed in this respect in order to have follow-up in terms of re-admissions for patients as this might show that appropriate management of patients with medical problems might prevent future admissions and thus save health care resources in the long term. Moreover, adequate screening of psychiatric patients for medical problems may identify those at risk for admission or readmission not only in a psychiatric but also in a medical ward. This should also be the focus of future research.

To an extent research on LOS has been replaced by research on determinants of health-care costs perhaps because early studies, though important, were not successful in identifying models that could account for a large variance in LOS. However, recent studies [14,15][34,35] are more successful and are in accordance with the results of this study in demonstrating how research on this topic can provide very useful insights into the management and improvement of psychiatric care of patients in the health care system.

The different ways in which inpatient care is organized in different contexts entails that the mean length of an acute psychiatric admission varies substantially. It should be stated that Greece has not developed primary care services. In this respect, some of the conditions resulting in specialist referral are ones that in another country one would normally expect to be managed in primary care. On the other hand, the population studied was admitted to an inpatients psychiatric setting and thus referral to secondary or primary care would not be applicable. The absence of further measures of psychopathology on admission and discharge is a limitation of this study. It is not expected that further standardised test for psychopathology would add to the validity of our findings. One cannot dismiss the possibility that detailed psychopathological testing would be of particular significance as it would permit us to investigate the effect of comorbidity on functioning and also the relationship between aspects of psychiatric pathology and comorbidity independent of LOS. Furthermore, since the physicians were aware that these patients were suffering from a psychiatric disorder one cannot altogether dismiss the claim that prolongation of admission was to an extent a result of a negative medical bias towards these patients (examinations took longer to complete, reviews were not as prompt as expected etc.).

## Conclusions

Our findings confirm previous studies which found high comorbidity among patients with bipolar disorder or schizophrenia. However, in the present study the rates were particularly elevated. Thus, an important aim of inpatient care remains the prompt assessment of comorbid physical illnesses of patients on admission, referral to other medical specialties during hospitalization and discharge of patients with specific guidelines and follow up appointments for reassessment. Moreover, our results offer concrete proof that lack of proper medical care for patients with schizophrenia whilst living in the community, is reflected in longer psychiatric stays. Although this phenomenon is not unknown to psychiatrists and other mental health professionals it remains unabated. Thus it results in more referrals for medical care and extended stay.

## Methods

### Design

This was a naturalistic study conducted in the adult inpatient psychiatric unit of the 2nd Psychiatry Department of the Athens University Medical School, which is based in Attikon General Hospital. Attikon Hospital serves the western suburbs of Athens which are the most densely populated area of the country. Data were collected from the medical and nursing files of the Department as well as the attending psychiatrists from February 2009 to February 2011. Measures of the study are detailed below. After the patients’ discharge we proceeded to analyze the data obtained. An inter-diagnosis design was used, comparing LOS and the other parameters studied in patients with schizophrenia and physical problems with patients with schizophrenia without physical problems and patients with bipolar illness and physical problems with bipolar patients without physical problems. Data were analyzed using PASW, version 18th.

Ethical approval for this research was granted by the Attikon University General Hospital ethics Committee and consequently by the Attikon General Hospital Scientific Committee. Due to the sensitive nature of this study in terms of both the information handled (access to medical notes, data, records) and the apparent severity of the mental health conditions involved, the participants were briefed and provided consent both on admission and after discharge. Shortly after admission patients were orally informed of the general aims of the study and were fully informed of their rights i.e. non-participation, withdrawal at any stage without consequences for their health care. In order not to unduly influence patients’ decisions to grant consent, a cooling off period of two months after discharge was decided by the researchers before each patient was re-approached to provide full informed oral and written consent. Thus, the sample presented here consists of the patients who freely provided oral and written informed consent, both on admission and after the cooling off period aforementioned.

### Participants

Participants were aged from 18 to 65. 180 of them had Greek nationality and 36 were non-Greeks. 200 suffered from schizophrenia (92 men and 108 women) and 228 from bipolar disorder (78 men and 150 women). Participants were either referred to the department inpatient unit as emergencies by mental health professionals or admitted via the emergency department of the hospital, which is on call for psychiatric patients twice a week. All patients were admitted voluntarily. No restraint, enforced medication or seclusion was used during the hospital admission of these patients.

### Procedure

Every patient admitted to the unit received a full physical examination and blood tests as per the units admission protocol (the blood tests included FBC, urea and electrolytes, cholesterol, liver and thyroid function test as well as a urine drug screen). If the results from these investigations pointed towards a problem then the appropriate referral was made. If the patient consistently complained of a somatic problem even without physical evidence and the problem was not resolved following a physical examination by the attending psychiatrist, then a specialist referral was also made. The number and type of referrals to other medical specialties was calculated by asking the attending psychiatrist of each patient and confirmed later by consulting the file of each patient.

Overall data were collected on: i) The sociodemographic characteristics of the patients on admission, ii) functioning and psychiatric symptom severity on admission and upon discharge and iii) comorbidity and type and number of referrals due to physical comorbidity on or during admission.

More specifically: Sociodemographic characteristics (age, sex, marital status, employment status during the past six years and during the six months prior to admission, urban or rural residence status). Psychiatric disorder characteristics (diagnosis, age of onset, number of prior hospitalizations, psychiatric comorbidity and substance misuse prior to admission).

Comorbidity requiring referral was a yes/no variable. As explained above, if a patient was referred to another medical specialty he would score a ‘yes’ answer. If the consulting specialist did not, on admission or during hospitalization, diagnose a medical problem that needed treatment, then the patient would score a ‘no’ answer. Thus, in the ‘no’ category we had patients 1) without concurrent medical condition or 2) without concurrent medical condition that needed further treatment. In the ‘yes’ category we had patients who had been diagnosed with a medical problem by the consulting specialist and received treatment for it.

We also noted the number and type of referrals to other medical specialties per patient in order to assess the prevalence of medical-surgical conditions that needed treatment in patients admitted with the diagnosis of either schizophrenia or bipolar disorder.

Classification of physical conditions was according to ICD-10 [36]. Thirteen broad categories of ICD-10 were used for an initial classification of the medical problems that the patient was referred for. Whenever the physician did not provide a diagnosis, then the patient would be categorized as not in need of medical intervention from the specialty he/she was referred to. If a patient was referred to the same medical speciality for more than one reason on the same referral, this would still count as one referral (this happened only on 3 occasions 1 male with schizophrenia and 2 patients with bipolar disorder (1 m, 1 f). We then re-categorized for specific physical conditions within the most commonly reported categories of our first categorization.

The social and psychological functioning of patients on admission and discharge was assessed by the Global Assessment of Functioning (GAF) scale. GAF is widely used in psychiatric practice and is the AXIS V of the DSM- IV-TR [37,38], it is easy to complete and has established good validity [39-41]. For the schizophrenia group we used one more scale: The Brief Psychiatric Rating Scale- Extended version (BPRS-E) [42] was used to assess symptom severity on admission for patients with schizophrenia. The BPRS-E is a psychometric instrument which comprises 24 symptom constructs, each rated in a 7-point scale of severity; it has been used extensively with patients with severe and persistent mental problems and has established good reliability and validity [43]. In addition, the BPRS-E total score has been a good predictor of LOS in patients with severe mental illness [30]. Finally, the Hamilton depression rating scale (HDRS) one of the most widely used scales for depression, with good reliability [44,45] and validity [46,47][48] was used for the patients with bipolar disorder.

## Competing interests

The authors declare that they have no competing interests.

## Authors’ contributions

AD was the principal investigator of the research study; he conceived and designed the study, coordinated the research process, wrote part of the paper and corrected the final draft. DS contributed to the conception and design of the study, coordinated the research, carried out the statistical analysis, wrote part of the paper and contributed to the final draft. SN, PN, AP, ENR, CC and CT collected the data and wrote part of the paper. DM wrote part of the paper and was responsible together with AD, DS and LL for the final draft. LL had the overall overseeing of the research, read and corrected the paper and was responsible together with AD and DS for ethical approval. All authors have read and approved the manuscript.

## Pre-publication history

The pre-publication history for this paper can be accessed here:

http://www.biomedcentral.com/1472-6963/12/166/prepub

## References

[B1] Lykouras L Douzenis A Do psychiatric departments in general hospitals have an impact on the physical health of mental patients? Curr Opin Psychiatry 2008 21 4 398 402 10.1097/YCO.0b013e32830079d0 18520746

[B2] Mitchell AJ Malone D Physical health and schizophrenia Curr Opin Psychiatry 2006 19 4 432 437 16721177 10.1097/01.yco.0000228767.71473.9e

[B3] McCreadie RG Diet, smoking and cardiovascular risk in people with schizophrenia: Descriptive study Br J Psychiatry 2003 183 6 534 539 10.1192/bjp.183.6.534 14645025

[B4] Muir-Cochrane E Medical co-morbidity risk factors and barriers to care for people with schizophrenia J Psychiatr Ment Health Nurs 2006 13 4 447 452 10.1111/j.1365-2850.2006.01002.x 16867129

[B5] Dixon L Postrado L Delahanty J Fischer PJ Lehman A The Association of Medical Comorbidity in Schizophrenia with Poor Physical and Mental Health TheJ Nerv Ment Dis 1999 187 8 496 502 10.1097/00005053-199908000-00006 10463067

[B6] Krishnan KRR Psychiatric and Medical Comorbidities of Bipolar Disorder Psychosom Med 2005 67 1 1 8 10.1097/01.psy.0000151489.36347.18 15673617

[B7] McIntyre RS Konarski JZ Soczynska JK Wilkins K Panjwani G Bouffard B Medical Comorbidity in Bipolar Disorder: Implications for Functional Outcomes and Health Service Utilization Psychiatr Serv 2006 57 8 1140 1144 10.1176/appi.ps.57.8.1140 16870965

[B8] Kilbourne AM Brar JS Drayer RA Xu X Post EP Cardiovascular Disease and Metabolic Risk Factors in Male Patients With Schizophrenia, Schizoaffective Disorder, and Bipolar Disorder Psychosomatics 2007 48 5 412 417 10.1176/appi.psy.48.5.412 17878500

[B9] Kemp DE Gao K Chan PK Ganocy SJ Findling RL Calabrese JR Medical comorbidity in bipolar disorder: relationship between illnesses of the endocrine/metabolic system and treatment outcome Bipolar Disord 2010 12 4 404 413 10.1111/j.1399-5618.2010.00823.x 20636638 PMC2913710

[B10] Lyketsos CG Dunn G Kaminsky MJ Breakey WR Medical Comorbidity in Psychiatric Inpatients: Relation to Clinical Outcomes and Hospital Length of Stay Psychosomatics 2002 43 1 24 30 10.1176/appi.psy.43.1.24 11927754

[B11] Mezzich JE Coffman GA Factors Influencing Length of Hospital Stay Hosp Community Psychiatr 1985 36 12 1262 1270 10.1176/ps.36.12.1262 4086000

[B12] Caton CLM Gralnick A A Review of Issues Surrounding Length of Psychiatric Hospitalization Hosp Community Psychiatr 1987 38 8 858 863 10.1176/ps.38.8.858 3111972

[B13] English J Sharfstein S Scherl D Astrachan B Muszynski I Diagnosis-related groups and general hospital psychiatry: the APA Study Am J Psychiatr 1986 143 2 131 139 3080906 10.1176/ajp.143.2.131

[B14] Hopko DR Lachar D Bailley SE Varner RV Assessing predictive factors for extended hospitalization at acute psychiatric admission Psychiatr Serv 2001 52 10 1367 1373 10.1176/appi.ps.52.10.1367 11585954

[B15] Blais MA Matthews J Lipkis-Orlando R Lechner E Jacobo M Lincoln R Predicting length of stay on an acute care medical psychiatric inpatient service Admin Pol Ment Health 2003 31 1 15 29 10.1023/A:1026044106172 14650646

[B16] Meyer JM Stahl SM The metabolic syndrome and schizophrenia Acta Psychiatr Scand 2009 119 1 4 14 10.1111/j.1600-0447.2008.01317.x 19133915

[B17] De Hert MA van Winkel R Van Eyck D Hanssens L Wampers M Scheen A Prevalence of the metabolic syndrome in patients with schizophrenia treated with antipsychotic medication Schizophr Res 2006 83 1 87 93 10.1016/j.schres.2005.12.855 16481149

[B18] Bermudes RA Keck PE Jr Welge JA The prevalence of the metabolic syndrome in psychiatric inpatients with primary psychotic and mood disorders Psychosomatics 2006 47 6 491 497 Schizophr Res 1 10.1176/appi.psy.47.6.491 17116950

[B19] O'Keane V Antipsychotic-induced hyperprolactinaemia, hypogonadism and osteoporosis in the treatment of schizophrenia J Psychopharmacol 2008 22 2 70 75 10.1177/0269881107088439 18477623

[B20] Aina Y Nandagopol J Nasrallah H Endocrine disorders in schizophrenia: relationship to antipsychotic therapy Curr Psychosis and Ther Rep 2004 2 2 78 83 10.1007/s11922-004-0035-4

[B21] Holt RIG Peveler RC Antipsychotics and hyperprolactinaemia: mechanisms, consequences and management Clin Endocrinol 2011 74 2 141 147 10.1111/j.1365-2265.2010.03814.x 20455888

[B22] Eaton WW Byrne M Ewald H Mors O Chen C-Y Agerbo E Association of Schizophrenia and autoimmune diseases: linkage of danish national registers Am J Psychiatr 2006 163 3 521 528 10.1176/appi.ajp.163.3.521 16513876

[B23] Poyraz BÇ Aksoy C BalcIoglu I Increased incidence of autoimmune thyroiditis in patients with antipsychotic-induced hyperprolactinemia Eur Neuropsychopharmacol 2008 18 9 667 672 10.1016/j.euroneuro.2008.04.014 18539008

[B24] Othman SS Kadir KA Hassan J Hong GK Singh BB Raman N High prevalence of thyroid function test abnormalities in chronic schizophrenia Aust New Zeal J Psychiatr 1994 28 4 620 624 10.3109/00048679409080785 7794205

[B25] Vonk R van der Schot AC Kahn RS Nolen WA Drexhage HA Is autoimmune thyroiditis part of the genetic vulnerability (or an Endophenotype) for bipolar disorder? Biol Psychiatr 2007 62 2 135 140 10.1016/j.biopsych.2006.08.041 17141745

[B26] Sloan DM Yokley J Gottesman H Schubert DSP A Five-Year study on the interactive effects of depression and physical illness on psychiatric unit length of stay Psychosom Med 1999 61 1 21 25 10024064 10.1097/00006842-199901000-00005

[B27] Schubert DSP Yokley J Sloan D Gottesman H Impact of the interaction of depression and physical illness on a psychiatric unit’s length of stay Gen Hosp Psychiatr 1995 17 5 326 334 10.1016/0163-8343(95)00065-Y 8522147

[B28] Jayaram G Tien A Sullivan P Gwon H Elements of a successful short-stay inpatient psychiatric service Psychiatr Serv 1996 47 4 407 412 8689373 10.1176/ps.47.4.407

[B29] Averill PM Hopko DR Small DR Greenlee HB Varner RV The role of psychometric data in predicting inpatient mental health service utilization Psychiatr Q 2001 72 3 215 235 10.1023/A:1010396831037 11467156

[B30] Anderson SW Crist AJ Payne N Predicting inpatient length of stay with the expanded version of the brief psychiatric rating scale (Version 4.0) Psychiatr Serv 2004 55 1 77 79 10.1176/appi.ps.55.1.77 14699205

[B31] Biancosino B Barbui C Grassi L The BPRS-E as predictor of length of stay in a residential facility Psychiatr Serv 2005 56 6 755 756 10.1176/appi.ps.56.6.755 15939961

[B32] Tzeng D-S Healthcare in schizophrenia: effectiveness and progress of a redesigned care network BMC Health Serv Res 2007 7 1 129 10.1186/1472-6963-7-129 17705853 PMC2000889

[B33] Geraty RD General hospital psychiatry and the new behavioral health care delivery system Gen Hosp Psychiatr 1995 17 4 245 250 10.1016/0163-8343(95)00038-S 7590187

[B34] Jimenez R Lam R Marot M Delgado A Observed-predicted length of stay for an acute psychiatric department, as an indicator of inpatient care inefficiencies. Retrospective case-series study BMC Health Serv Res 2004 4 1 4 10.1186/1472-6963-4-4 15102334 PMC387834

[B35] Stevens A Hammer K Buchkremer G A statistical model for length of psychiatric in-patient treatment and an analysis of contributing factors Acta Psychiatr Scand 2001 103 3 203 211 10.1034/j.1600-0447.2001.00043.x 11240577

[B36] WHO ICD-10 international statistical classification of diseases and related health problems 2007 Geneva: World Health Organization

[B37] American Psychiatric Association Diagnostic and statistical manual of mental disorders: DSM-IV-TR. 4., text revision 2000 Washington: American Psychiatric Association

[B38] Aas IHM Guidelines for rating Global Assessment of Functioning (GAF) Ann Gen Psychiatr 2011 10 1 2 12 10.1186/1744-859X-10-2 PMC3036670 21251305

[B39] Mirandola M Baldassari E Beneduce R Italo A Segala M Tansella M A standardized and reliable method to apply the Global Assessment of Functioning (GAF) scale to psychiatric case records Int J Meth Psychiatr Res 2000 9 2 79 86 10.1002/mpr.82

[B40] Startup M Jackson MC Bendix S The concurrent validity of the Global Assessment of Functioning (GAF) Br J Clin Psychol 2002 41 4 417 422 10.1348/014466502760387533 12437796

[B41] Jones S Thornicroft G Coffey M Dunn G A brief mental health outcome scale-reliability and validity of the Global Assessment of Functioning (GAF) Br J Psychiatr 1995 166 5 654 659 10.1192/bjp.166.5.654 7620753

[B42] Dingemans P Linszen D Lenior M Smeets R Component structure of the expanded Brief Psychiatric Rating Scale (BPRS-E) Psychopharmacology 1995 122 3 263 267 10.1007/BF02246547 8748395

[B43] Burlingame GM Seaman S Johnson JE Whipple J Richardson E Rees F Sensitivity to change of the Brief Psychiatric Rating Scale-Extended (BPRS-E): An item and subscale analysis Psychol Serv 2006 3 2 77 87

[B44] Hamilton M A rating scale for depression J Neurol Neurosurg Psychiatr 1960 23 56 62 10.1136/jnnp.23.1.56 14399272 PMC495331

[B45] Hamilton M Development of a rating scale for primary depressive illness Br J Soc Clin Psychol 1967 6 4 278 296 10.1111/j.2044-8260.1967.tb00530.x 6080235

[B46] Morriss R Leese M Chatwin J Baldwin D Inter-rater reliability of the Hamilton depression rating scale as a diagnostic and outcome measure of depression in primary care J Affect Disord 2008 111 2–3 204 213 18374987 10.1016/j.jad.2008.02.013

[B47] Furukawa TA Assessment of mood: Guides for clinicians J Psychosom Res 2010 68 6 581 589 10.1016/j.jpsychores.2009.05.003 20488276

[B48] Zheng Y Zhao J Phillips M Liu J Cai M Sun S Validity and reliability of the Chinese Hamilton Depression Rating Scale Br J Psychiatr 1988 152 5 660 664 10.1192/bjp.152.5.660 3167442

